# Harvest selection on Atlantic cod behavioral traits: implications for spatial management

**DOI:** 10.1002/ece3.244

**Published:** 2012-07

**Authors:** Esben Moland Olsen, Michelle R Heupel, Colin A Simpfendorfer, Even Moland

**Affiliations:** 1 Institute of Marine Research FlødevigenN-4817 His, Norway; 2 Centre for Ecological and Evolutionary Synthesis (CEES), Department of Biology, University of OsloP.O. Box 1066 Blindern, N-0316 Oslo, Norway; 3 Australian Institute of Marine ScienceTownsville, Queensland 4811, Australia; 4 Fishing and Fisheries Research Centre, School of Earth and Environmental Sciences, James Cook UniversityTownsville, Queensland 4811, Australia

**Keywords:** Atlantic cod, behavior, harvesting, personality trait, selection, telemetry

## Abstract

Harvesting wild populations may contrast or reinforce natural agents of selection and potentially cause evolutionary changes in life-history traits such as growth and maturation. Harvest selection may also act on behavioral traits, although this field of research has so far received less attention. We used acoustic tags and a network of receivers to monitor the behavior and fate of individual Atlantic cod (*Gadus morhua*, *N* = 60) in their natural habitat on the Norwegian Skagerrak coast. Fish with a strong diel vertical migration, alternating between shallow- and deep-water habitats, had a higher risk of being captured in the fishery (traps, gillnet, hand line) as compared to fish that stayed in deeper water. There was also a significant negative correlation between fish size (30–66 cm) and the magnitude of diel vertical migration. Natural selection on behavior was less clear, but tended to favor fish with a large activity space. On a monthly time scale we found significant repeatabilities for cod behavior, meaning that individual characteristics tended to persist and therefore may be termed personality traits. We argue that an evolutionary approach to fisheries management should consider fish behavior. This would be of particular relevance for spatial management actions such as marine reserve design.

## Introduction

It is now widely recognized that human harvesting of wild animals and plants may have evolutionary impacts on the targeted species within a contemporary time frame (Law and Salick 2005; Hendry et al. 2008; Hutchings and Fraser 2008; Conover et al. 2009). Harvesting can be intense and selective for particular phenotypes (Coltman et al. 2003; Carlson et al. 2007; Nusslé et al. 2009), so that humans as predators may outpace natural agents of trait change (Edeline et al. 2007; Darimont et al. 2009). There is growing concern that such harvest-induced evolution may have long-term negative impacts on the productivity of harvested populations (Walsh et al. 2006; Kuparinen and Merilä 2007; Allendorf and Hard 2009; Dunlop et al. 2009a). The mechanism behind adaptive evolution is selection. Therefore, there is a clear applied value to understanding the strength and direction of harvest selection and how this scales with natural selection (Law 2000; Carlson et al. 2007). In some cases harvest selection and natural selection can act in the same direction (Swain 2011), while in other cases they will act in opposite directions (Carlson et al. 2007; Olsen and Moland 2011).

Studies on fisheries-induced evolution have focused mainly on changes in life-history traits related to body size, such as growth and maturation schedules (Rijnsdorp 1993; Olsen et al. 2004; Swain et al. 2007; Nusslé et al. 2011). The potential for an evolutionary response in behavioral traits has been less studied but could be expected, especially in fisheries using passive gears (traps, gillnets, hook, and line), where behavioral characteristics such as boldness and activity level could directly influence the probability of encountering fishing gear (Uusi-Heikkilä et al. 2008). Indeed, experimental angling of largemouth bass (*Micropterus salmoides*) led to an evolutionary response in angling vulnerability after only four generations of selection (Philipp et al. 2009). This response was linked to various physiological and behavioral characteristics, as well as growth rate (Cooke et al. 2007; Redpath et al. 2009, 2010). Furthermore, experimental gillnetting of rainbow trout (*Oncorhynchus mykiss*) documented rapid depletion of genotypes with fast growth and bold personality traits from the harvested populations (Biro and Post 2008; see also Askey et al. 2006). The fact that behavior can be genetically correlated with life-history traits linked to productivity (Chiba et al. 2007) means that selection on either of these traits could reduce the potential for recovery of overharvested populations.

In aquatic animals, a common behavior is to alternate between shallow-water occupancy during dark hours and deep-water occupancy during light hours (Zaret and Suffern 1976). From an evolutionary perspective, such diel vertical migration is typically explained as a trade-off between foraging and predator avoidance, where food-rich shallow habitats are exploited in the shelter of darkness (Gliwicz 1986; Clark and Levy 1988). Also, diel vertical migration may represent a thermoregulatory strategy allowing ectotherms to adjust their metabolic rates by moving between cold deep waters and warmer surface layers (Wurtsbaugh and Neverman 1988; Sims et al. 2006). In any case, there is potential for human-induced selection to interfere with such a naturally shaped behavioral pattern, if, for instance, fishing intensity is nonrandom with regard to depth. Diel vertical migrations are also relevant for the protection of fish stocks within marine reserves, because habitat shifts may involve both vertical and horizontal movements (Sims et al. 2006).

Marine reserves are considered a promising tool for conservation and fisheries management (Gaines et al. 2010). So far, most of the research effort on marine reserves has focused on demographic responses to protection (Russ et al. 2008; Goñi et al. 2010). Removing fishing mortality from certain areas may also have evolutionary consequences for the managed populations. On one hand marine reserves could help to counter fisheries-induced evolution of life-history traits (Baskett et al. 2005; Dunlop et al. 2009b; Miethe et al. 2010). On the other hand, such areas may set up new selection pressures on fish behavior, favoring individuals that tend to have restricted movement and thus stay within reserve boundaries (Parsons et al. 2010).

Detecting harvest selection and natural selection on fish behavior in the wild can be a challenging task. However, acoustic monitoring offers new opportunities in this respect because it potentially allows for continuous monitoring of individual movements and fates (Hightower et al. 2001; Pine et al. 2003). Each study animal is equipped with an acoustic tag transmitting a unique signal to a network of moored receiver stations. Tags may also have a pressure sensor so that both horizontal and vertical movements can be tracked. In species such as blacktip shark (*Carcharhinus limbatus*) and Atlantic cod (*Gadus morhua*), natural mortality has been inferred from cessation of movement within a study area, while harvest mortality has been inferred from disappearance of individuals inside the monitoring network in combination with tag returns from fishers (Heupel and Simfendorfer 2002; Olsen and Moland 2011).

In this study, we used acoustic monitoring to quantify selection gradients from both harvesting and natural mortality on cod behavioral traits in their natural habitat on the Norwegian Skagerrak coast. On a small scale (3 km^2^), we show that fish with a strong diel vertical migration have a higher risk of being harvested compared to fish that utilize deeper locations. Behavior and life history was linked in the sense that smaller fish tended to have a stronger diel vertical migration compared to larger fish. We discuss our results in relation to the implementation of evolutionarily enlightened fisheries management. In particular, our results are relevant for incorporating this aspect into design of marine reserves.

## Materials and Methods

### Study species

**Figure 1:**
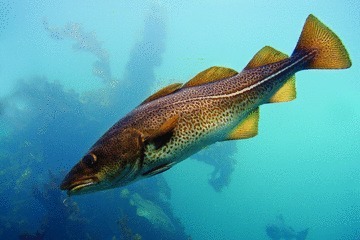
Atlantic cod (*Gadus morhua*). Photograph by Øystein Paulsen used with permission.

The Atlantic cod (Fig. 1) is an ecologically and commercially important species found in coastal and offshore shelf habitats in the eastern and western North Atlantic Ocean. Cod are highly fecund and spawn multiple batches of pelagic offspring within an elongated spawning season (Sars 1865; Kjesbu 1989). In Skagerrak, the age 0 juveniles settle in shallow-water nursery areas during May and June (Dahl and Dannevig 1906), grow about 10–15 cm per year and mature at an age of 2–4 years and a body length of 30–50 cm (Dannevig 1954; Olsen et al. 2008). Coastal Skagerrak holds a network of local cod populations (Knutsen et al. 2003, 2011). Low dispersal and spawning in sheltered fjord basins protected from coastal currents are likely mechanisms maintaining this fine-scale population structure (Dannevig 1954; Espeland et al. 2008; Ciannelli et al. 2010). Coastal cod in Skagerrak are caught by commercial and recreational fishers using a wide variety of gears, such as hand line, traps, long line, and gillnet (Julliard et al. 2001).

### Study system

**Figure 2:**
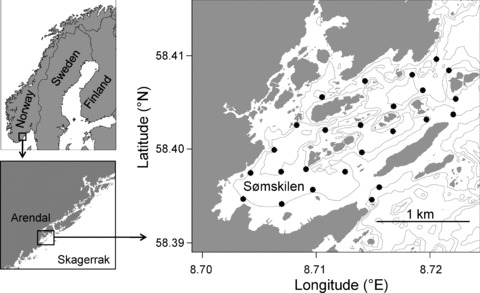
Sømskilen study area south off the town of Arendal on the Norwegian Skagerrak coast, showing the network of 25 acoustic monitoring receivers (black dots) used to detect and store signals transmitted from tagged Atlantic cod. Gray lines indicate the 5, 10, and 20 m depth contours.

This study was conducted on the Norwegian Skagerrak coast, near the town of Arendal (Fig. 2). The study area (Sømskilen bay) is a 3 km^2^ semi-sheltered basin with several small islands. Shallow (1–5 m) habitats consist of rocky habitat dominated by macroalgae or soft bottom with eel grass beds (Espeland et al. 2010). Deeper habitats (down to 30 m) consist mainly of mud flats. Within the study area cod typically perform diel vertical migrations, utilizing shallow habitats during night and retreating to deeper areas during daytime (Espeland et al. 2010).

### Tagging and monitoring

Wild cod were captured in the Sømskilen basin in May 2008 using fyke nets. Candidates for tagging were measured to the nearest centimeter (fork length) and brought to the Flødevigen marine research station (Institute of Marine Research) about 5 km northeast of the study area. A total of 60 fish were selected for tagging, based on body length and position of capture within the study area.

For acoustic monitoring of cod behavior and fate, the fish were equipped with V9P-2L transmitters (9 × 38 mm, weight in seawater < 3 g, Vemco Division, Amirix Systems Inc., Halifax, Canada). This tag has a built in pressure sensor (accuracy ± 2.5 m when deployed at max. 50 m depth) and transmits the current depth along with an identity code for each tag. Transmitters were programmed to transmit a signal every 110–250 sec, with random intervals to reduce code collision (i.e., instances of two or more tags simultaneously transmitting to one receiver). Estimated battery life of these transmitters was 660 days. Transmitters were surgically implanted in the abdominal cavity while fish were anesthetized with clove oil (Munday and Wilson 1997). The wound was closed using absorbable suture material. Additionally, all cod equipped with acoustic transmitters received an external T-bar anchor tag (TBA-2, 45 × 2 mm, Hallprint Pty. Ltd, Holden Hill, South Australia) parallel to the anterior dorsal fin. These tags had printed information posting a reward of 500 NOK if returned to the Institute of Marine Research.

Tagged fish were observed in large aquaria for 1–3 days between tagging and release to ensure full recovery from the surgical procedure. All fish were released during daytime in shallow water (1–5 m depth, 22–28 May) at the position of initial capture. There was no tagging mortality. All fish looked healthy and immediately oriented themselves toward the bottom when released. A total of 25 ultrasonic receivers (VR2W, Vemco Division, Amirix Systems Inc.) were deployed in the study area to record signals emitted from transmitters. Receivers were deployed at 2 m depth, attached to surface buoys moored throughout the area from 9 May 2008 (Fig. 2). In Sømskilen there are three channels through which fish can leave the study area. In each of these we placed two sentinel receivers to record any passage of tagged cod. Data were downloaded from VR2W receivers at sea at least once every second month, and stored in a VUE data base (Vemco Division, Amirix Systems Inc.).

### Data analyses

The presence and movement of fish within the receiver network was determined from detections at multiple receivers and depths through time. A range testing study showed that the network provided very good coverage of the study area, with overlapping detection ranges among multiple receivers (Olsen and Moland 2011). This means that as long as a fish was alive and moving within the study area, we ought to have detected it. Based on these tracking data and tag recoveries from fishers, the fate of each fish was classified on monthly intervals as either (1) harvested within the study area, (2) died within the study area, (3) survived within the study area, or (4) dispersed out of the study area (Heupel and Simpfendorfer 2002; Olsen and Moland 2011). A fish was classified as harvested when the high-reward T-bar tag was returned and the fish was no longer detected within the receiver array. A fish was classified as dead (but not harvested) when there was no longer any logged data showing horizontal and vertical movement (usually with continued signals from a fixed depth within the study area) and no high-reward tag was reported. A fish was classified as alive within the study area when multiple detections indicated horizontal and vertical movement. A fish was considered to have left the study area when detections indicated directional movement toward the outermost receivers (bordering the open ocean), followed by absence of detections for the rest of the study. The study lasted for 18 months (end of May 2008 to beginning of December 2009), after which the receiver network was retrieved.

To quantify cod behavior, we first used an algorithm developed by Simpfendorfer et al. (2002) to estimate the mean horizontal position (latitude and longitude) of each fish during consecutive 30 min time intervals based on the logged receiver data. Within an array of receivers with partly overlapping detection ranges, the algorithm estimates a mean position of an animal as the mean position of the receivers weighted by the number of detections at each receiver (Simpfendorfer et al. 2002). Thus, the method does not provide an exact location of an animal at a given time, but rather estimates short-term (30 min) centers of activity. This approach was applied because individual detections will not represent the exact position of the fish, but will simply indicate that the fish was within listening range of the receiver. The vertical location was determined as mean depth for the same 30 min time intervals. Diel vertical migration was estimated as the daily depth range (maximum observed depth–minimum observed depth). Horizontal swimming pattern was estimated as the linearity of movement during five consecutive centers of activity locations. First, all centers of activity locations were converted to Universal Transverse Mercator (UTM) measures to provide distance measures in meters. Next, the distance between steps one and five was calculated and divided by the total distance (sum of each of the five steps) to produce a ratio of linearity where values approaching 1 indicate linear movement (Simpfendorfer et al. 2010). Hence, this linearity measure was intended to estimate the tendency of a fish to move consistently in one direction. The pattern of horizontal swimming was also quantified as the standard deviation of linearity, indicating the degree of horizontal behavior shifts (i.e., a high standard deviation of linearity suggests that the fish often changed behavior between high and low directionality). Lastly, the horizontal and vertical positions were used as input variables for calculating monthly three-dimensional kernel utilization distributions, using an approach recently developed by Simpfendorfer et al. (2012). This method estimates the volumetric space use of animals based on tracking data. For species that experience a three-dimensional environment, such as the cod, this approach will yield a more comprehensive representation of animal movement compared to traditional two-dimensional measures. In total, we quantified five aspects of cod behavior: *vertical position* (daily depth), *diel vertical migration* (daily depth range), *horizontal movement* (linearity of swimming), *horizontal shifts* (standard deviation of linearity), and *activity space* (three-dimensional kernel utilization distribution). These metrics formed the basis for our analyses on harvest selection and natural selection in this coastal system. For the analyses, each metric was calculated on a monthly basis for each individual (average of depth, daily depth range, and linearity). Specifically, a working hypothesis was that harvest selection would act directly on depth use (vertical position and diel vertical migration), since part of the fishing activity in the area is typically conducted close to land (fyke nets and hand line; E. M. Olsen, pers. obs.).

Diel vertical migration is presumably an evolved strategy linked to predation and/or thermoregulation (see Introduction), and natural selection on these behavioral traits could differ from harvest selection in strength and direction. Harvest selection could also act on horizontal movement pattern. Specifically, Allendorf and Hard (2009) suggested that harvesting could selectively remove individuals with a more predictable behavior. We explored this hypothesis by testing whether linearity of movement influenced the probability of surviving the fishery. More generally, mechanisms determining a fish's vulnerability to fishing gear are still not well understood (Redpath et al. 2009). Quantifying selection on horizontal movement pattern could help to elucidate such mechanisms and generate hypotheses for future studies. Lastly, activity space served as a measure of the broad-scale activity level of the cod. A working hypothesis was that a larger activity space could increase the vulnerability to capture by fishing gear. Prior to the statistical analyses, all behavioral traits were ln-transformed to stabilize the variance.

We used a standard logistic regression approach to estimate selection acting on cod behavioral traits (Lande and Arnold 1983; Janzen and Stern 1998). Survival (*s*) was used as response variable, corresponding to an absolute fitness of one (survived) or zero (harvested or dead from other causes). To facilitate comparison with other studies, all predictor variables were standardized to a mean of zero and a standard deviation of one. Our starting model (prior to model selection) had the following structure:


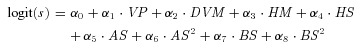


and thus included slope parameters (α) for vertical position (*VP*), diel vertical migration (*DVM*), horizontal movement (*HM*), horizontal shifts (*HS*), and activity space (*AS*). A squared effect of activity space (*AS*^2^) was included to test for stabilizing (negative parameter) or disruptive (positive parameter) selection on this trait. We also added a linear and nonlinear effect of body size (*BS*, *BS*^2^), in accordance with previous findings (Olsen and Moland 2011). Logistic regression coefficients (α) from the most parsimonious model (see Results) were transformed to obtain approximate selection gradients (β_avggrad_) on a relative fitness scale using the method of Janzen and Stern (1998). Selection gradients estimate the strength of selection acting directly on each trait independent of correlations with other traits in the model (Brodie et al. 1995). Through univariate regressions we also estimated selection differentials: the direct selection acting on each trait plus any indirect selection caused by correlations with other traits. Quadratic regression coefficients were multiplied with 2 to obtain nonlinear selection gradients (Stinchcombe et al. 2008).

We first modeled the probability of surviving the fishery (i.e., harvest selection). In this analysis, all fish defined as dispersed or dead from natural causes were censored to facilitate a direct comparison between fish that were either harvested or survived (see also Olsen and Moland 2011). Next, we modeled the probability of surviving from natural agents of selection. Here, all dispersed and harvested fish were censored. Model selection was based on the Akaike's Information Criterion corrected for small sample size (AIC_C_) as defined by Hurvich and Tsai (1989). We compared the AIC_C_ score of a restricted set of models rather than testing all possible combinations of predictor variables (Burnham and Anderson 1998). Sample size also limited the number of predictor variables that could be included.

Due to rapid depletion of the study population caused mostly by harvesting (see Results) the statistical analyses focused mainly on what happened during the first summer (June–September) after tagging, while sample size was still adequate (*N* = 56). Specifically, we used June 2008 as the common observation period for estimating behavioral traits. Next, we used these trait estimates as predictor variables when modeling cod fates during the following 3-month interval (July–September). Three months were considered a compromise between accumulating a sufficient number of fates and not moving too far in time from the point where the behavior and life-history trait (body size) was measured (Olsen and Moland 2011). We tested for correlations (Pearson correlation coefficients) among behavioral traits in June, and between body size and each of the behavioral traits. All data analyses were performed using program R (version 2.11.1, R Development Core Team 2010).

We used the multiple (monthly) individual measurements to estimate repeatability of cod behavior. In general, repeatability is defined as the proportion of the total variance in multiple measurements of a trait that is due to differences among individuals (Falconer and Mackay 1996). For two measures of the same trait, repeatability can be calculated as the regression coefficient for the second measure on the first measure (Dohm 2002). The concept of repeatability is of relevance to evolutionary biology because it can elucidate what is the upper bound to heritability (Falconer and Mackay 1996), although this interpretation may not always be accurate (Dohm 2002). In any case repeatability will inform about the consistency of individual cod behavioral traits measured in our study. Specifically, we explored whether the behavior in June would be a reasonable indicator of behavior during the following 3-month period when selection was estimated. We did three separate regressions for each trait because sample size changed during the study (due to mortality): July against June, August against June, and September against June. Repeatability was estimated as the slope parameter for each of these regressions (Dohm 2002). This provided us with three consecutive estimates of repeatability for each trait.

## Results

**Figure 3:**
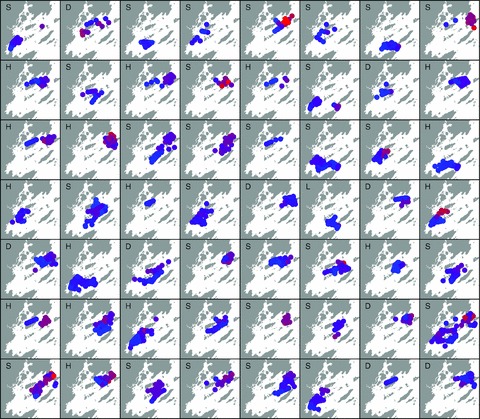
Positions of individual Atlantic cod during June 2008 estimated from acoustic monitoring. Color denotes logged depth, ranging from 0 m (blue) to 30 m (red). Letters refer to the fate of each individual during July–September 2008: S = survived, H = harvested, D = died, and L = left the area. For map scale and details, see Figure 1.

Four fish were removed from the dataset before analyzing behavioral traits due to sparse or lacking data from the common observation period in June (i.e., early cases of dispersal and mortality). The remaining 56 individuals ranged from 30 to 66 cm in body length (mean = 45 cm). During the following 3-month period (July–September), 29% of these fish were known to be harvested, while an additional 16% died and one individual dispersed, leaving 54% of the fish alive within the study area (Fig. 3). As reported by Olsen and Moland (2011), mortality and dispersal accumulated further after this so that by the end of the 18-month study duration, none of the cod were left alive and transmitting within the study area. Both commercial fishers and recreational fishers reported that they had harvested tagged cod. Commercial harvest was by gillnets and traps, while recreational catch was also by hand line.

**Figure 4:**
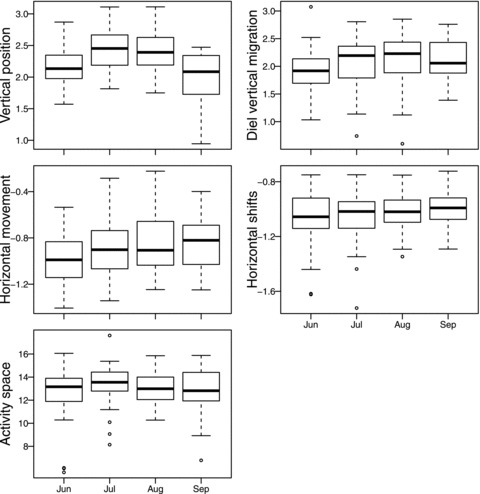
Box plots of Atlantic cod behavioral traits during June–September 2008, showing vertical position (meter depth), diel vertical migration (meters), horizontal movement pattern (linearity), horizontal movement shifts (SD of linearity), and three-dimensional activity space (m^3^). All variables are shown on a log scale.

In June, the mean vertical position occupied by individual cod ranged from 4.8 to 17.6 m while the mean diel vertical migration ranged from 2.8 to 21.6 m (Fig. 4; see also Fig. 3). The estimated activity space ranged from 312 to 9.420.852 m^3^ (Fig. 4). There was a significant positive correlation between diel vertical migration and activity space, and between horizontal movement and horizontal shifts (*P* < 0.05; Table 1). There was also a significant negative correlation between vertical position and horizontal movement, between vertical position and horizontal movement shifts, and between horizontal movement shifts and activity space (*P* < 0.05; Table 1). There was a significant negative correlation between fish body length and diel vertical migration (*P* = 0.0087; Table 1), while none of the other correlations between fish body size and behavioral traits were significant (*P* > 0.35; Table 1).

**Table 1:** Correlations among behavioral traits (VP: vertical position, DVM: diel vertical migration, HM: horizontal movement, HS: horizontal shifts, AS: activity space) and life-history trait (BS: body size) of Atlantic cod. Prior to the analyses, all variables were standardized to a mean of zero and a standard deviation of unity

Trait	VP	DVM	HM	HS	AS	BS
VP	1	0.045	−0.29	−0.36	0.24	−0.13
DVM	0.045	1	0.18	0.030	0.36	−0.35
HM	−0.29	0.18	1	0.67	0.066	−.082
HS	−0.36	0.030	0.67	1	−0.47	0.081
AS	0.24	0.36	0.066	−0.47	1	−0.080
BS	−0.13	−0.35	−0.082	0.081	−0.080	1

Model selection based on AIC_C_ supported additive effects of vertical position, diel vertical migration, horizontal shifts, and body size on the probability of surviving the fishery (Table 2). Specifically, harvesting selected against fish that utilized shallow depths and displayed extensive diel vertical migration, while favoring fish that displayed extensive horizontal shifts in movement (Table 3). As reported previously (Olsen and Moland 2011), harvesting also selected against large fish (Table 3). Adding an effect of activity space to the model increased the AIC_C_ by 2.1 units and hence there was little support for this effect (Table 2). Removing the effects of vertical position and horizontal movement shifts from the model only increased the AIC_C_ by 1.5 units (Table 2), indicating that these simpler models also had some support. However, to provide estimates of selection we relied on the model with the lowest AIC_C_ score (Burnham and Anderson 1998). Selection differentials estimated from simple regressions were smaller than the selection gradients estimated from the multiple regression, but pointed in the same direction (vertical position: α = 0.54, SE = 0.33, β_avggrad_ = 0.18; diel vertical migration: α = –0.26, SE = 0.32, β_avggrad_ = –0.09; horizontal shifts α = 0.24, SE = 0.33, β_avggrad_ = 0.08; body size: α = –0.67, SE = 0.33, β_avggrad_ = –0.21). In terms of natural agents of selection, our modeling (Table 2) supported a weak positive effect of activity space (Table 3). As reported previously (Olsen and Moland 2011), there was also some support for nonlinear natural selection on body size (Table 3). Note that two other candidate models could not be clearly separated from the best model (difference in AIC_C_ < 2) and that the null model (no effects) did not differ from the top model by more than 3.5 AIC_C_ units (Table 2). To facilitate a direct comparison of harvest selection and natural selection, we also applied the most parsimonious harvesting model to the data on natural mortality. None of the predictor variables were significant (*P* > 0.14), but, with the exception of body size, the estimated selection gradients tended to point in the same direction as those in the harvesting model (vertical position: α = 0.65, SE = 0.45, β_avggrad_ = 0.14; diel vertical migration: α = –0.24, SE = 0.42, β_avggrad_ = –0.05; horizontal shifts: α = 0.24, SE = 0.41, β_avggrad_ = 0.05; body size: α = 0.32, SE = 0.48, β_avggrad_ = 0.07).

**Table 2:** Logistic regression modeling of Atlantic cod survival in a coastal habitat, with separate analyses of harvest selection and natural selection and showing the model structure, deviance (Dev), number of parameters (P), and AIC_C_ value. Models in bold, having the lowest AIC_C_ value, were used for estimating selection gradients. Explanatory variables include vertical position (*VP*), diel vertical migration (*DVM*), horizontal movement pattern (*HM*), horizontal movement shifts (*HS*), activity space (*AS*), and body size (*BS*). For details, see Materials and Methods

Model no.	Model structure	Dev	P	AIC_C_
Harvest selection				
1	*VP*+*DVM*+*HM*+*HS*+*AS*+*AS*^2^+*BS*+*BS*^2^	41.46	9	64.46
2	*VP*+*DVM*+*HM*+*HS*+*AS*+*AS*^2^+*BS*	42.53	8	62.42
3	*VP*+*DVM*+*HM*+*HS*+*AS*+*BS*	42.97	7	59.92
4	*VP*+*DVM*+*HS*+*AS*+*BS*	44.75	6	58.91
**5**	***VP*+*DVM*+*HS*+*BS***	**45.28**	**5**	**56.78**
6	*VP*+*DVM*+*BS*	49.41	4	58.40
7	*DVM*+*BS*	51.71	3	58.28
8	*BS*	54.94	2	59.22
9	NULL	59.44	1	61.53
Natural selection				
1	*VP*+*DVM*+*HM*+*HS*+*AS*+*AS*^2^+*BS*+*BS*^2^	25.60	9	49.81
2	*VP*+*VS*+*HM*+*HS*+*AS*+*BS*+*BS*^2^	26.05	8	46.85
3	*VP*+*HM*+*HS*+*AS*+*BS*+*BS*^2^	26.72	7	44.33
4	*VP*+*HM*+*AS*+*BS*+*BS*^2^	28.63	6	43.25
5	*VP*+*AS*+*BS*+*BS*^2^	29.63	5	41.45
**6**	***AS*+*BS*+*BS*^2^**	**31.58**	**4**	**40.75**
7	*BS*+*BS*^2^	34.71	3	41.40
8	*BS*	41.42	2	45.75
9	NULL	42.14	1	44.24

**Table 3:** Harvest selection and natural selection acting on Atlantic cod behavior and life history, showing logistic regression coefficients (α) with standard errors (SE) and also the approximate selection gradients (β_avggrad_) as defined by Janzen and Stern (1998)

Variable	α	SE	P	β_avggrad_
Harvest selection				
Vertical position (*VP*)	0.796	0.408	0.051	0.198
Diel vertical migration (*DVM*)	−0.866	0.419	0.039	−0.215
Horizontal shifts (*HS*)	0.900	0.466	0.054	0.224
Body size (*BS*)	−1.065	0.445	0.017	−0.265
Natural selection				
Activity space (*AS*)	0.833	0.503	0.098	0.144
Body size squared (*BS*^2^)	24.597	12.837	0.055	8.526

**Figure 5:**
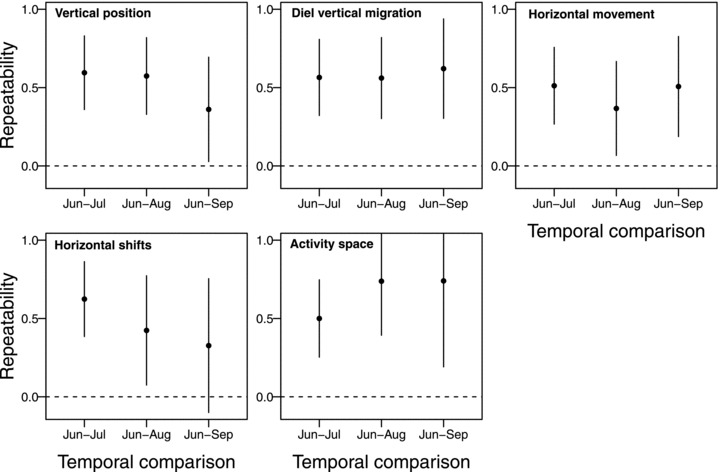
Repeatabilities for individual Atlantic cod behavioral traits across three temporal comparisons (June–July, June–August, and June–September), showing the mean prediction and 95% confidence interval.

When testing for consistent individual differences in behavior, we found that all between-month repeatabilities of behavioral traits were positive and significantly different from zero (*P* < 0.05), with one exception for the comparison between horizontal movement shifts in June and September (Fig. 5). The overall mean repeatability (across traits and months) was 0.53 (SD = 0.13).

Lastly, we evaluated the consistency of these selection gradients by applying the best model to data from a second time interval, that is, July behavior as predictor of fate during August–October. Sample size limitations (*N* < 30) caused by accumulated cod mortality restricted any further comparisons. In relation to harvesting, selection gradients based on July behavior pointed in the same direction as those based on June behavior, although the depth effect was less clear (vertical position: α = 0.20, SE = 0.45, β_avggrad_ = 0.06; diel vertical migration: α = –1.42, SE = 0.61, β_avggrad_ = –0.42; horizontal shifts: α = 0.83, SE = 0.58, β_avggrad_ = 0.24; body size: α = –1.02, SE = 0.50, β_avggrad_ = –0.30). The selection gradients relating to natural agents of selection also appeared fairly consistent (activity space: α = 0.32, SE = 0.48, β_avggrad_ = 0.06; body size squared: α = 27.95, SE = 13.29, β_avggrad_ = 5.25).

## Discussion

Our study on coastal Atlantic cod provides empirical support to the expectation of direct selection on behavioral traits in fisheries that operate with passive gear such as trapping, angling, and gill netting (Heino and Godø 2002; Uusi-Heikkilä et al. 2008). Harvesting selected against a diel vertical migration behavior and favored fish that remained in deeper waters. Harvesting also selected against fish with a consistent horizontal movement pattern and fish with a large body size. Furthermore, we provide a direct comparison of harvest selection and natural selection, where directional natural selection on behavior appeared comparatively weak. Cod behavior was correlated with life history in the sense that smaller fish tended to display more extensive diel vertical migrations than larger cod. When combined in the same model, both behavioral traits and body size had a significant influence on the probability of surviving the fishery. The selection gradients estimated from this multiple logistic regression model were larger than the selection differentials estimated from simple logistic regression models on the same traits. This suggests that (1) selection acted directly on both the behavior and the life history of this fish, and (2) the correlation between the behavior and life history acted to reduce the selection intensity. This is because selection gradients estimate the strength of selection acting directly on each trait independent of correlations with other traits in the model, while selection differentials estimate the direct selection acting on each trait plus any indirect selection caused by correlations with other traits. In our case, the tendency for smaller fish to display more pronounced diel vertical migrations probably caused them to be more exposed to the fishery and thereby reducing (but not removing) the fisheries selection against large body size. We discuss the relevance of our results for the development of a Darwinian (evolutionary) perspective on the management of harvested populations, with special reference to the design of marine no-take reserves.

Acoustic monitoring in combination with high-reward external tags is a powerful tool for quantifying fish behavior and fate (Hightower et al. 2001; Pine et al. 2003), but some uncertainty nevertheless remains. For instance, the impact of harvesting may have been underestimated if external tags were lost or if fishers did not claim the 500 NOK reward. Using a double-tagging approach, Cadigan and Brattey (2006) estimated the retention rate of external T-bar tags placed near the posterior end of the first dorsal fin (as was the case in our study) on Atlantic cod to be about 0.85 for the first 100 days after tagging and release. In our study, we kept the fish for observation for 1–3 days after tagging and did not detect any tag loss during this period. Furthermore, a broad scale tagging study conducted along the Skagerrak coast since 2005 (Espeland et al. 2008), using the same tags, ensured the majority of fishers were aware of the possibility of catching a tagged cod without targeting telemetry-tagged fish in particular. As shown by Baker et al. (2011), there could also be significant selection due to delayed mortality associated with dis-entanglement from fishing gear. In total, this suggests that we have provided a conservative estimate on the impact of harvesting. There were no instances where a fisher claimed the reward while the fish continued to move within the study area, which would indicate that the external tag had been removed and the fish subsequently released. Therefore, reports represent actual harvest events. We note that our sample size is fairly limited (60 fish) and hence is not likely to detect weak, but biologically significant, selection. The sample size was defined by the cost of the acoustic tags, not our effort in the field or the availability of fish. Also, we present selection estimates from 1 year, while in many cases the strength and form of selection will vary among years (Siepielski et al. 2009).

The fact that we used fyke nets to capture the study animals could have influenced our results. This passive gear may have biased our initial sample toward relatively active individuals. Probably, our selection estimates are conservative since potentially less active fish (not in our sample) would also likely be less exposed to other passive fishing gear used by the local fishers. In any case, there was relatively large variation in behavioral traits within our sample on which selection could act, and indeed acted.

Shallow near shore habitats such as eelgrass beds and kelp forests often have a rich fauna of prey for cod (Lekve et al. 1999; Fredriksen et al. 2010). Studies from the Norwegian Skagerrak coast have shown that cod will exploit these areas during the night (Espeland et al. 2010) and feed on shore crabs, shrimps, polychaetes, gobies, and a variety of other organisms (Hop et al. 1992). It is perhaps not surprising that fishing is concentrated in what may be the prime cod feeding habitats on this coastline. However, if diel vertical migration behavior has a heritable component, then ongoing harvest selection could ultimately drive the cod population toward a more stationary, deep-resident behavior, with potential negative effects on harvest yield. A recent study on cod from Iceland found evidence for a genetic difference between shallow-water and deep-water cod, and furthermore, that fisheries selected strongly against shallow-water genotypes (Árnason et al. 2009). The authors present genotypic fitness estimates that predict rapid disappearance of shallow-water fish around Iceland. Our study differs from the Icelandic case by directly monitoring small-scale behavior, but together the two studies indicate that harvest selection against shallow-water occupancy may be a widespread phenomenon and therefore should be considered in fisheries management. The combined effect of harvesting on behavior and life histories is also relevant for anadromous and terrestrial systems. For example, there is evidence for directional selection from gillnet fishing acting on behavior (timing of spawning migration) as well as life history (age and size at maturity) in sockeye salmon (Quinn et al. 2007; Kendall et al. 2009). Furthermore, Sasaki et al. (2008) found evidence for evolutionary responses in life-history traits as well as antipredator behavior of Japanese snakes hunted for medical and nutritional value.

The selective impact of natural mortality on depth use and diel vertical migration appeared less strong and was not significant, although the selection gradients tended to point in the same direction as for harvest selection. Therefore, harvest selection might act to reinforce, rather than contrast, the natural trade-off associated with this behavior (Clark and Levy 1988). However, our data does not allow for any strong conclusions on this point.

There was a phenotypic correlation between cod behavior and life history, where smaller cod displayed more pronounced diel vertical migrations compared to larger cod. This pattern could be due to within-generation selection against shallow-water excursions, meaning that the surviving bigger fish were those with a more stationary deep-resident behavior. In support of this, we found significant repeatabilities for depth use and diel vertical migration on a 1–3 month time frame, showing that individual characteristics tended to persist. In addition, natural trade-offs associated with diel vertical migration behavior could depend on fish body size.

The significant repeatabilities of cod behavioral characteristics imply that these can be termed personality traits. According to Dall et al. (2004), animal personality traits can be defined as consistent individual differences in behavior in time and/or across contexts. Diel vertical migration may represent a trade-off between feeding opportunity and risk of predation (Clark and Levy 1988), and fish that actively seek shallow habitats might therefore be classified as bold. Our study therefore points in the same direction as that of Biro and Post (2008), showing that fishing may remove bold personalities from harvested populations. The significant repeatabilites also imply that fish behavioral traits during June 2008, which we used as predictor variables in the statistical models, was indeed representative of their behavior during the following period when selection was estimated (July–September 2008).

Allendorf and Hard (2009) hypothesized that harvesting could select against individuals with predictable movement patterns. Our data provide some support for this hypothesis. First, we have shown that diel vertical migrations (predictable behavior) involve an increased risk of being captured in the fishery. Second, we found that fish with a more variable horizontal movement pattern (unpredictable behavior; shifting between linear and nonlinear movements) had a better chance of surviving the fishery. The mechanisms behind this latter finding remain unclear, but we offer the result as a starting point, which could guide hypotheses in future studies.

Spatial restrictions on fishing gear use could help to counter harvest selection on fish behavior, for instance by limiting the use of traps in shallow water. Still, there is a risk that such restrictions would simply alter the direction of fisheries selection and not solve the problem. Marine reserves represent another management tool that could help to preserve naturally shaped life histories and behavioral patterns. Selection against diel vertical migration and shallow-water occupancy could be countered by including both shallow and deep habitats within reserve boundaries. On the other hand, marine reserves may also create new challenges. In our study, we show that while there was a tremendous amount of variation in activity space among individuals, harvest selection on this trait appeared weak. Introducing closed areas would expectedly change this and favor those individuals which do not move beyond reserve borders (Parsons et al. 2010). One reason for this is the effect known as “fishing the line” where fishers concentrate effort along the borders of marine reserves (Kellner et al. 2007). The idea that reserve effectiveness depends on size is supported empirically by a meta-analysis of data from European reserves, in which population density in reserves increased with reserve size, suggesting that larger reserves protect a greater proportion of mobile fish (Claudet et al. 2008). To account for potentially detrimental fishery driven evolution with regard to space use, future reserve networks should ideally be designed to account for most of the observed variation in movement behavior of targeted species.

In conclusion, our study provides an example of why an evolutionary approach to fisheries management should also consider the spatial ecology of harvested species, and how spatial management actions need to be carefully designed to protect naturally shaped behavioral patterns.
